# Single- and multi-trait genomic prediction and genome-wide association analysis of grain yield and micronutrient-related traits in ICARDA wheat under drought environment

**DOI:** 10.1007/s00438-023-02074-6

**Published:** 2023-10-18

**Authors:** Wuletaw Tadesse, Zakaria El Gataa, Fatima Ezzahra Rachdad, Adil El Baouchi, Zakaria Kehel, Admas Alemu

**Affiliations:** 1 The International Center for Agricultural Research in the Dry Areas (ICARDA), Rabat, Morocco; 2 AgroBioSciences, Mohammed VI Polytechnic University (UM6P), Ben Guerir, Morocco; 3 Department of Plant Breeding, Swedish University of Agricultural Sciences, Alnarp, Sweden

**Keywords:** Bread wheat, Micronutrients, GWAS, Multi-trait genomic prediction

## Abstract

Globally, over 2 billion people suffer from malnutrition due to inadequate intake of micronutrients. Genomic-assisted breeding is identified as a valuable method to facilitate developing new improved plant varieties targeting grain yield and micronutrient-related traits. In this study, a genome-wide association study (GWAS) and single- and multi-trait-based genomic prediction (GP) analysis was conducted using a set of 252 elite wheat genotypes from the International Center for Agricultural Research in Dry Areas (ICARDA). The objective was to identify linked SNP markers, putative candidate genes and to evaluate the genomic estimated breeding values (GEBVs) of grain yield and micronutrient-related traits.. For this purpose, a field trial was conducted at a drought-prone station, Merchouch, Morocco for 2 consecutive years (2018 and 2019) followed by GWAS and genomic prediction analysis with 10,173 quality SNP markers. The studied genotypes exhibited a significant genotypic variation in grain yield and micronutrient-related traits. The GWAS analysis identified highly significantly associated markers and linked putative genes on chromosomes 1B and 2B for zinc (Zn) and iron (Fe) contents, respectively. The genomic predictive ability of selenium (Se) and Fe traits with the multi-trait-based GP GBLUP model was 0.161 and 0.259 improving by 6.62 and 4.44%, respectively**,** compared to the corresponding single-trait-based models. The identified significantly linked SNP markers, associated putative genes, and developed GP models could potentially facilitate breeding programs targeting to improve the overall genetic gain of wheat breeding for grain yield and biofortification of micronutrients via marker-assisted (MAS) and genomic selection (GS) methods.

**Supplementary Information:**

The online version contains supplementary material available at 10.1007/s00438-023-02074-6.

## Introduction

Wheat is the world's leading food crop and the cradle of human civilization with an average annual production of 770 million tons in about 220 million hectares (FAOSTAT 2021). It has been playing a fundamental role in improving global food security by providing an average of 19% of calories and 21% of the protein needed for daily human diets (Shewry 2009). In addition to carbohydrates and proteins, wheat grains also contain several essential micronutrients, such as iron (Fe), zinc (Zn), and selenium (Se), which play a significant role in human growth and development, cognitive and immune function, and gene regulation. However, reports have shown that more than 2 billion people worldwide, mainly women and children in developing countries, suffer from Fe and Zn malnutrition (Lim et al. 2013). This leads to anemia, stunted physical growth, neuron motor retardation, fatigue, and reduced productivity (Umamaheswari et al. 2011). At the global level, 43% of children, 38% of pregnant and 29% of non-pregnant women of reproductive ages are anemic (World Health Organization 2015). Although diversification of diets, provision of mineral supplements, and food fortification are potential solutions, none of these approaches is sustainable for solving micronutrient malnutrition (Gletsu-Miller and Wright 2013). Identification, development, and deployment of wheat varieties with high micronutrients content is the most economical, feasible, and sustainable approach to prevent malnutrition, especially in the developing world (Shewry 2009; Alomari et al. 2018; Rathan et al. 2022). Genomic-assisted breeding techniques including QTL mapping via GWAS and recently genomic prediction are widely applied methods to assist the development of new crop varieties with an improved micronutrient content.

Climate change reduces rainfall, exacerbates water stress, and has a significant negative impact on wheat production. Drought induced by climate change affects wheat grain production and quality at any growth stage and reduces wheat grain production up to 30% (Lesk et al. 2016). In addition to increasing yield and improve stress tolerance/ resistance, enhancing wheat quality under drought conditions has been identified as the key objective of wheat breeding programs (Suliman et al. 2021). Research findings have shown that wheat wild relatives are identified as an important source for micronutrients and tolerance/resistance for many biotic and abiotic stresses. Many efforts have been carried out to transfer these genes into adapted cultivars through the utilization of synthetic hexaploid wheat through the hybridization of tetraploid wheat (2*n* = 4*x* = 28, AABB) with the diploid wild relative, *Aegilops tauschii* (2*n* = 2*x* = 14, DD) followed by chromosome doubling of the corresponding diploids (Li et al. 2018). Synthetic hexaploids are great sources for improving wheat qualities including macronutrients and micronutrients and other economically relevant traits (Tadesse et al. 2015). According to recent reports, more than 30% of the elite spring bread wheat genotypes in the CIMMYT and ICARDA breeding programs contain synthetic wheat of different percentages in their pedigrees (Tadesse et al. 2019). Understanding the genetic variation and dissecting the genetic basis of traits in these elite germplasms is very important to further improve wheat quality traits, especially Fe and Zn composition in breeding programs.

Previous studies using biparental populations have reported QTLs for Fe concentrations on wheat chromosomes 6A, 3B, 7B, and 4D (Wang et al. 2021). Marker-trait associations (MTAs) linked to Fe and Zn concentrations in wheat grains have also been reported through GWAS analysis (Tadesse et al. 2019). In addition to GWAS, genomic prediction (GP), also called genomic selection (GS) when applied in practical breeding to select individual plants, have been applied to determine the genome-estimated breeding values (GEBVs) of quantitatively inherited traits from the genomic data of selection candidates without undertaking field phenotyping or expensive quality analysis (Alemu et al. 2021; Alomari et al. 2018). The GEBVs of candidate or breeding individuals are determined using genomic prediction models developed from the genotypic and phenotypic data of the training population with several statistical models (Goiffon et al. 2017).

The current study was carried out using 252 elite spring bread wheat genotypes developed from ICARDA with the following objectives: (I) to evaluate the available genotypic variation in micronutrient content and grain yield; (II) to identify significantly linked MTAs with targeted traits via GWAS and localize putative candidate genes; and (IV) to estimate the GEBVs of target traits through single-trait- and multi-trait-based genomic prediction analysis. For this purpose, the panel was evaluated for 2 consecutive years (2018 and 20,219) under field condition and genotyped with 15 K SNP array.

## Materials and methods

### Plant material and field experimental conditions

The current study used a panel comprising 252 elite wheat genotypes developed from ICARDA (Supplementary Table S1). The field experiment was carried out at the ICARDA experimental station in Merchouch, Morocco (33°36′24.3"N 6°42′50.0"W, 430 masl) for two consecutive cropping seasons (2017–2018 and 2028–2019). The field trial was conducted testing all genotypes in a 3 m^2^ plot size following an alpha lattice design with two replications. Planting was done on the first week of December during the two trial seasons similarly at a seeding rate of 100 kg per hectare. Merchouch station is characterized with a cambisol soil and was received an annual precipitation of about 320 mm over the two cropping seasons. Merchouch station has a moderate humidity with annual temperature ranging between 10 and 40 °C.

### Phenotyping of grain yield and micronutrients

The currently used panel was phenotyped for four traits including grain yield (GY), iron (Fe), zinc (Zn), and selenium (Se). The micronutrient content of 252 elite wheat samples was evaluated following the standard protocol explained by Pequerul et al. (1993). Briefly, a total of 0.5 g of whole grain flour sample was placed in a digestion tube (QBlock series, Horiba) and kept overnight after adding 8 ml of nitric acid (HNO3). Samples were exposed for heating at 90 °C for 60 min followed by adding 3–4 ml of 30% hydrogen peroxide (H_2_O_2_). Then samples were filtered followed by diluting with hydrochloric acid (HCl) after the digestion process was completed and the solution became colorless. The determination of micronutrient content was performed using a simultaneous multi-element inductively coupled plasma emission spectrometer (iCAP-7000Duo, Thermo Fisher Scientific) at the Cereal and Legume Quality Laboratory, ICARDA, Morocco. Grain yield was measured in kg/plot after threshing the whole plot.

The ANOVA, standard deviation as well as the coefficient of variation was determined using the *RcmdrMisc* package in R (Fox et al. 2022). The broad-sense heritability of traits was estimated using the formula:

$$H^{2} = {\text{Vg}}/{\text{Vp}}$$

where *H*^2^ represents the broad-sense heritability, Vg is the genotypic variance, and Vp is the phenotypic variance. The PCA and biplot analysis of phenotypic data for the four traits were conducted using the *FactoMineR* in R environment.

### DNA extraction and genotyping

Genomic DNA was extracted from fresh leaf samples of 2-week-old seedlings following the procedures described by Ogbonnaya et al. (2001). Genotyping was carried out using the 15 K SNP array at SGS Institute Fresenius GmbH, Trait Genetics in Gatersleben, Germany. A total of 10,173 SNP markers were applied for the current genomic prediction, linkage disequilibrium, population structure, and marker-trait association analysis after filtering out below-standard-quality SNP markers with less than 5% minor allelic frequency (MAF) and above 20% missing values per individual.

### Linkage disequilibrium and population structure analyses

The pairwise SNP markers linkage disequilibrium (LD) was calculated as *r*^2^ using Trait Analysis by Association, Evolution, and Linkage (TASSEL) software (Bradbury et al. 2007) with a full size making a total of 786,219,856 marker comparisons. The LD decay trend through generations was estimated using the procedure developed by Remington et al. (2001) in R language following the nonlinear regression model developed by Hill and weir (Hill and Weir 1988) with the formula:

$$E\left( {r^{2} } \right) = \left[ {\frac{{10 + C^{{}} }}{{\left( {2 + C} \right)\left( {11 + C} \right)}}} \right] \left[ {1 + \frac{{\left( {3 + C} \right)\left( {12 + 12C + C^{2} } \right)}}{{n\left( {2 + C} \right)\left( {11 + C} \right)}}} \right]$$

where *E*(*r*^2^) is the expected value of *r*^2^ under drift-recombination equilibrium; *c* is the recombination fraction between sites and *n* is the sample size.

### Genome-wide association analysis and gene annotation

Genome-wide association analysis of the current panel from the absolute mean values of grain yield and micronutrient traits was performed using the mixed linear model (Q + K) in TASSEL including the kinship matrix (K) and principal components (Q) to control possible false-positive results raised from kinship and population structure, respectively. Manhattan plots were generated to display significant markers across chromosomes using *CMplot* package in the R environment using −log10(p) > 3.0 as exploratory threshold (LiLin-Yin 2018). The physical position of SNP markers was retrieved from the International Wheat Genome Sequence Consortium (IWGSC) v1.1 (IWGS 2018). The Ensemble Plants database (https://plants.ensembl.org/Triticum_aestivum/Info/Index) was explored to identify putative functional genes-associated SNP markers identified as MTAs with studied traits following the variant effect predictor method, and the UniProt (https://www.uniprot.org) database was used to determine associated protein functions.

### Single- and multi-trait genomic prediction and cross-validation analysis

Both the single- and multi-trait genomic prediction analysis was conducted using the genomic best unbiased prediction (GBLUP) model. The single-trait GBLUP-based genomic prediction analysis was done following the mixed linear model formula:

$$Y = 1\beta + {\text{Z}} u + \varepsilon$$

where Y is the single-trait phenotypic observations of wheat genotypes for yield and micronutrient traits, β is the overall mean, Z is the design matrix for the random effects of SNP markers, *u* is the vector of additive effects derived from SNP markers following the multivariate normal μ∼*MN* (**0**, **G**σ^2^_g_), where **G** is the marker-based genomic relationship matrix calculated using the method developed by VanRaden (2008) as $G = \frac{ZZ^{\prime}}{p}$, where Z is the centered and standardized SNP marker matrix and p is the number of markers and σ^2^_g_ is the genetic variance, ε is the vector of the error term derived similarly following the multivariate normal (**0**, **I**σ^2^_e_), where **I** is the identity matrix and σ^2^_e_ is the residual variance.

The micronutrient-related traits genomic prediction analysis was further analyzed with multi-trait based genomic prediction models following the mixed model formula:

$$\left[ {\begin{array}{*{20}c} {Y_{1} } \\ {Y_{2} } \\ {Y_{3} } \\ \end{array} } \right] = \left[ {\begin{array}{*{20}c} {\beta_{1} } \\ {\beta_{2} } \\ {\beta_{3} } \\ \end{array} } \right] + \left[ {\begin{array}{*{20}c} {Z_{1 } } & 0 & 0 \\ 0 & {Z_{2 } } & 0 \\ 0 & 0 & {Z_{3 } } \\ \end{array} } \right]\left[ {\begin{array}{*{20}c} {{ }u_{1} } \\ {{ }u_{2} } \\ {{ }u_{3} } \\ \end{array} } \right] + \left[ {\begin{array}{*{20}c} {\varepsilon_{1} } \\ {\varepsilon_{2} } \\ {\varepsilon_{3} } \\ \end{array} } \right]$$

where *Y*_*ij*_* i* = 1, 2, 3, and *j* = 1, …, n is a matrix of the response of the _i_th trait related to micronutrients in the _j_th wheat genotype, *β*_1_- *β*_3_ is a matrix from intercepts of each of the three micronutrient-related traits, *Z*_1_- *Z*_3_ the matrices of random SNP markers effect, *u*_1_—*u*_3_ is a matrix of vector derived from markers effect, and *ε*_1_ – *ε*_3_ is a matrix of the error terms from each traits.

A fivefold cross-validation analysis was employed to evaluate the single- and multi-trait genomic prediction models where 80% of the panel was used to develop the prediction model. The developed model was used to estimate the genomic breeding values (GEBVs) of the remaining 20% individuals. The prediction accuracy was derived from the correlation between the GEBVs with recorded phenotypic values of yield and micronutrient-related traits. The cross-validation analysis was done for 100 iterations.

All the single- and multi-trait genomic prediction analysis was done in BGLR software package (Pérez and Los 2014) computed with Markov Chains Monte Carlo (MCMC) sampler with chain length of 12,000 iterations and 10 thinning intervals with the first 2000 was used as burn-in.

## Results

### Phenotypic analysis

**Fig. 1:**
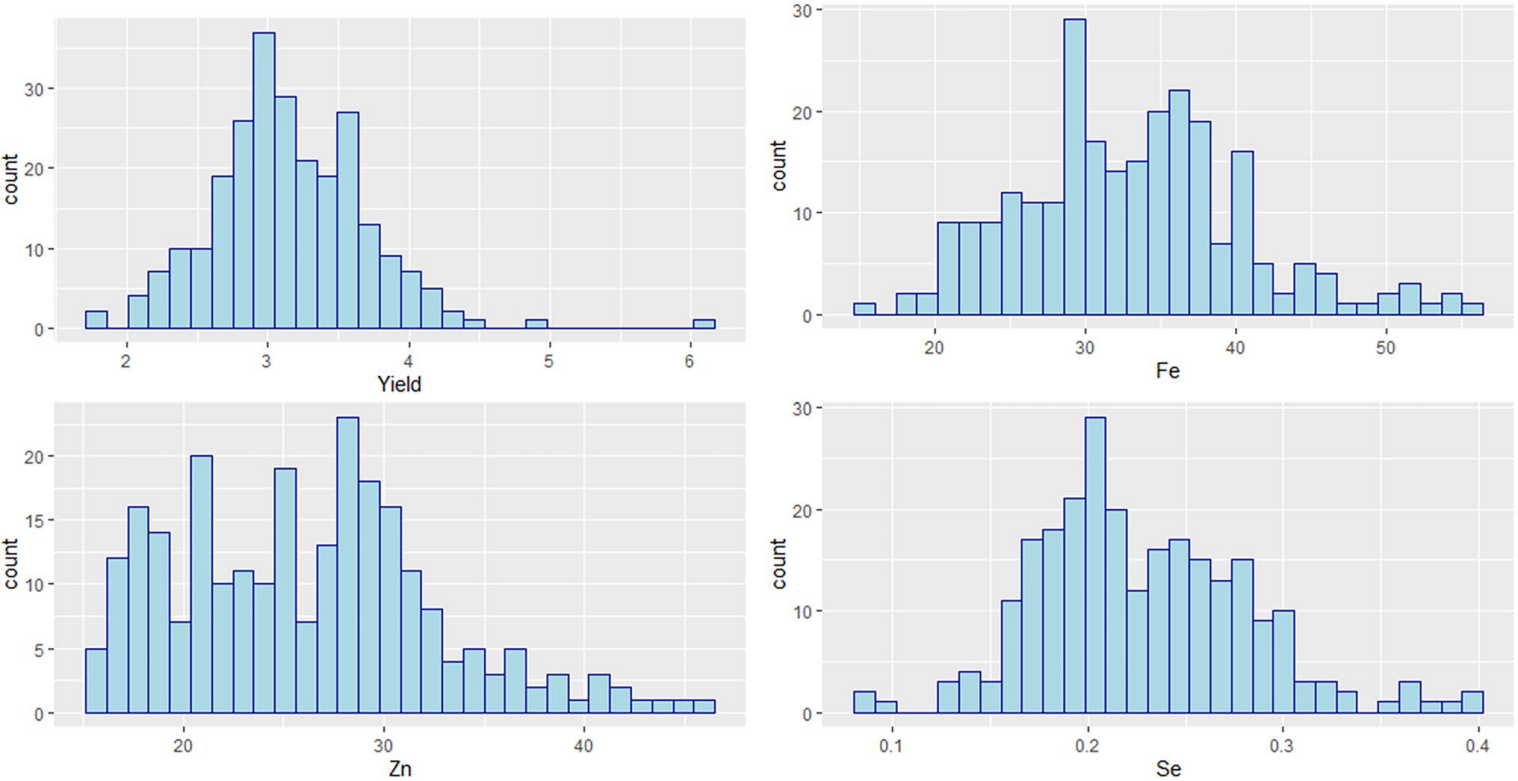
Frequency distribution for grain yield and micronutrient-related traits recorded from the 252 elite wheat genotypes tested for 2 years in Merchouch, Morocco

The highly performed elite genotypes for grain yield can be found in Supplementary Table S1 with their pedigree information. The two genotypes G-163 and G-233 showed the highest grain yield scoring with 6.13 and 4.85 t/ha, respectively. A normal frequency distribution was observed from the phenotypic values for all four studied traits (Fig. 1). Elite lines showed a significant genotypic variation with a wide range from 1.82 to 6.13 t/ha with an average 3.16 t/ha while the broad-sense heritability was 0.42 (Table 1). All the micronutrient-related traits showed a significant variation among genotypes. Elite genotypes exhibited a wide range of variation for Zn, Fe, and Se with a score ranging from 15.33 to 45.62, 15.68 to 56.06, and 0.08 to 0.39 and with average values of 26.04, 45.62, and 0.22 mg/kg, respectively. The Se trait exhibited the highest broad-sense heritability with 0.68 followed by Fe and Zn contents with 0.64 and 0.47, respectively, while GY had showed only 0.39.

**Table 1:** Analysis of variance for grain yield and micronutrient-related traits from 252 elite wheat genotypes recorded from 2 years field trials in Merchouch

Trait	Min	Max	Mean	Sd	CV	H^2^
Fe	15.68	56.06	32.95	7.44	22.7	0.64
Zn	15.33	45.62	26.13	6.5	24.8	0.47
Se	0.08	0.39	0.22	0.05	23.7	0.68
GY	1.82	6.13	3.16	0.54	17.2	0.39

*H*^2^ broad-sense heritability, *Sd* standard error, *CV* coefficient of variation

### Pearson correlation and principal component analyses

**Fig. 2:**
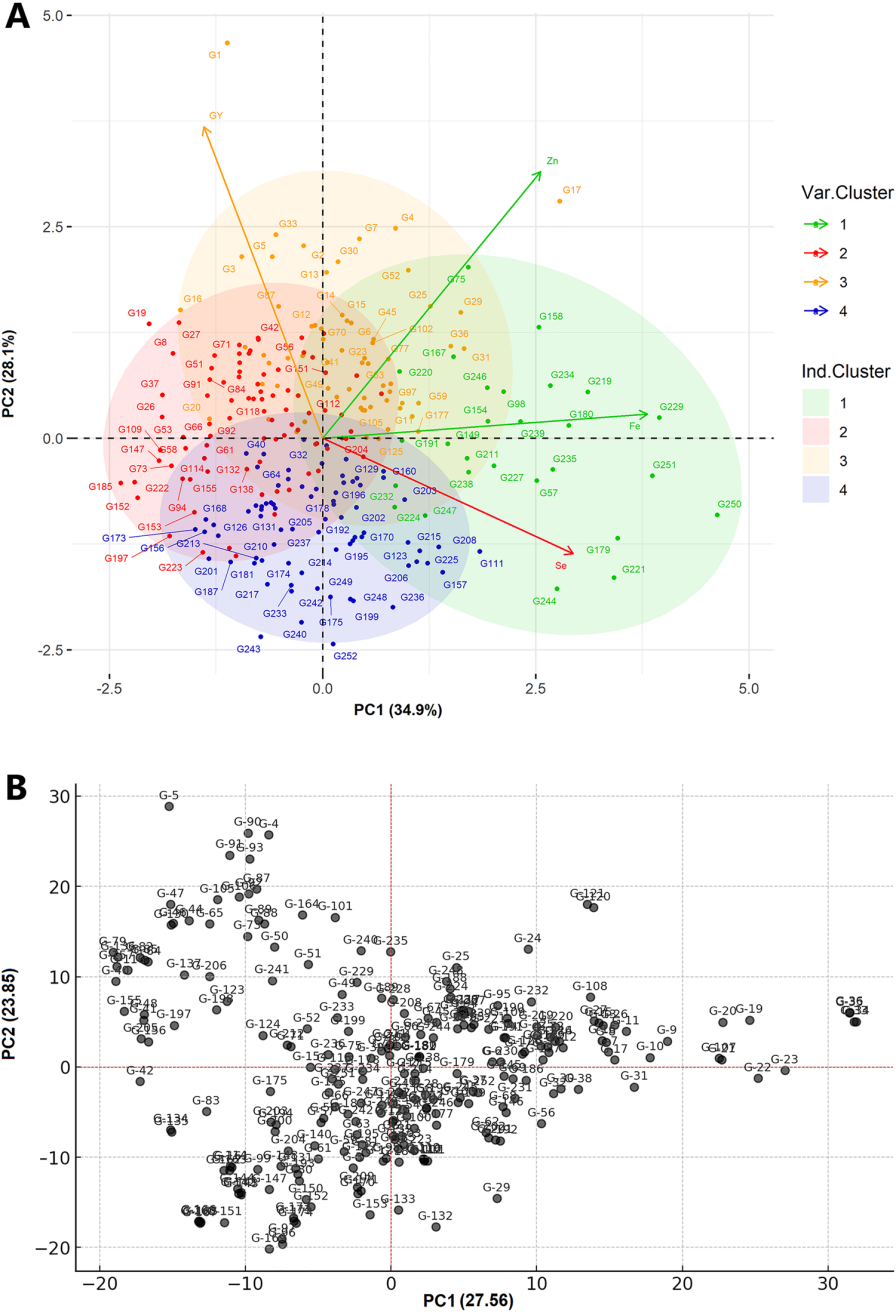

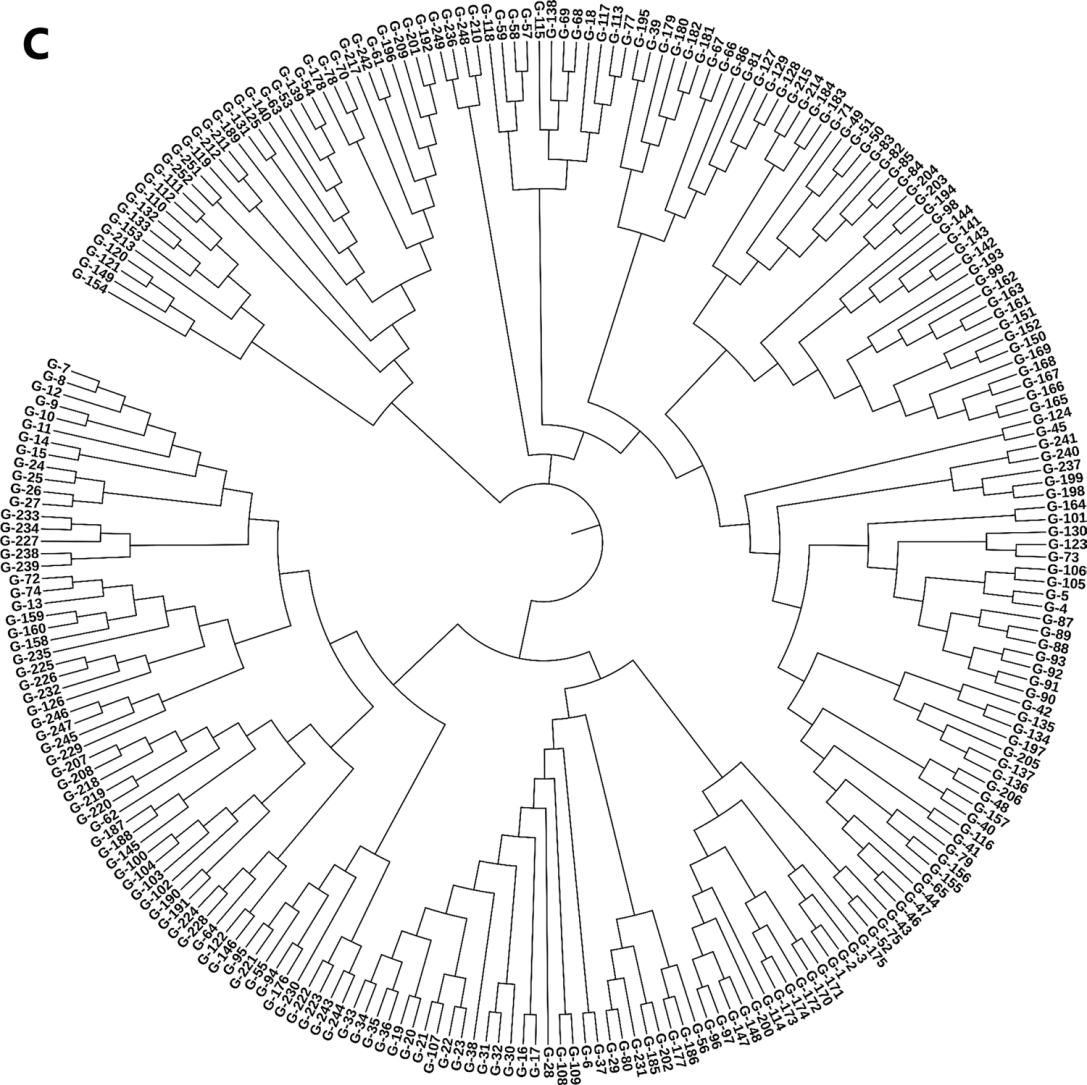
Cluster and principal component biplot analysis of tested bread wheat genotypes in Merchouch (Morocco). Biplot analysis of genotypes according to their phenotypic performance (**A**), principal component (**B**) and neighbor_joining tree (NJ) analysis (**C**) of the 252 genotypes with their SNP marker profile

**Fig. 3:**
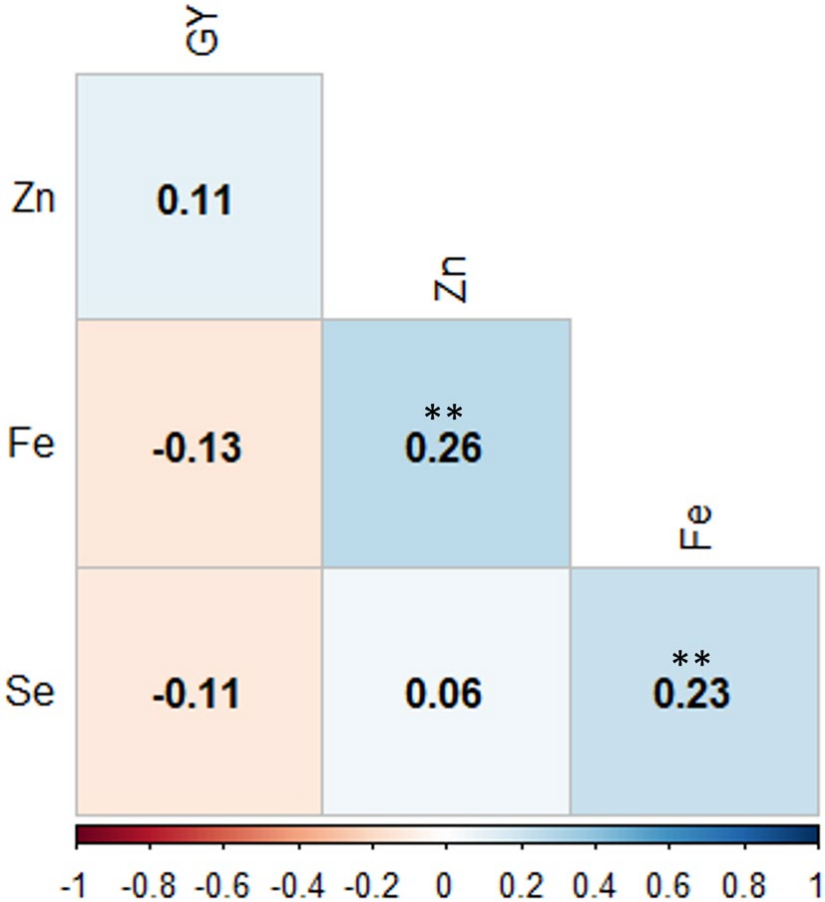
Pearson’s correlation between grain yield and the three micronutrient-related traits. **Significant at *p* < 0.01

Principal component analysis (PCA) with phenotypic scorings clustered the 252 genotypes into 4 distinct groups (Fig. 2). The first and second principal components accounted for 34.9% and 28.1% of the total phenotypic variation observed in grain yield and micronutrient-related traits, respectively. Eigenvalue for cluster one was trended to the right while clusters two, three, and four were located to the left, top, and bottom side from the biplot origin, respectively. The vectors of the biplot analysis for Fe and Zn were moved on the positive direction above the origin. In contrast, the vector value for grain yield was above the origin but in the left direction, while the vector for Se was below the origin and in the right direction. Fe and Zn contents were grouped in cluster 1, Se in cluster 2, and grain yield grouped in cluster 3. The SNP markers files with the neighbor-joining (NL) tree and principal component algorithms grouped elite lines with their respective pedigree similarity (Fig. 2b and c). For instance, the genotypes G-1, G-2, and G-3 clustered together on the NJ tree are linked to each other as sister lines with the same pedigree (CROC-1/AE.SQUARROSA(224)//OPATA/4/SERI.1B*2/3/KAUZ*2/BOW//KAUZ) while the G-142 (SERI.1B*2/3/KAUZ*2/BOW//KAUZ/4/ATTILA/3*BCN) and G-143 (SERI.1B*2/3/KAUZ*2/BOW//KAUZ/4/TRAP#1/BOW//PFAU/3/MILAN) are sharing the same node in their pedigree information (Supplementary Table S1). Furthermore, Fe showed a significant and positive correlation with Zn and Se with Pearson’s correlation coefficients r^2^) 0.23 and 0.26, respectively (Fig. 3).

### Linkage disequilibrium, marker-trait association, and gene annotation

**Fig. 4:**
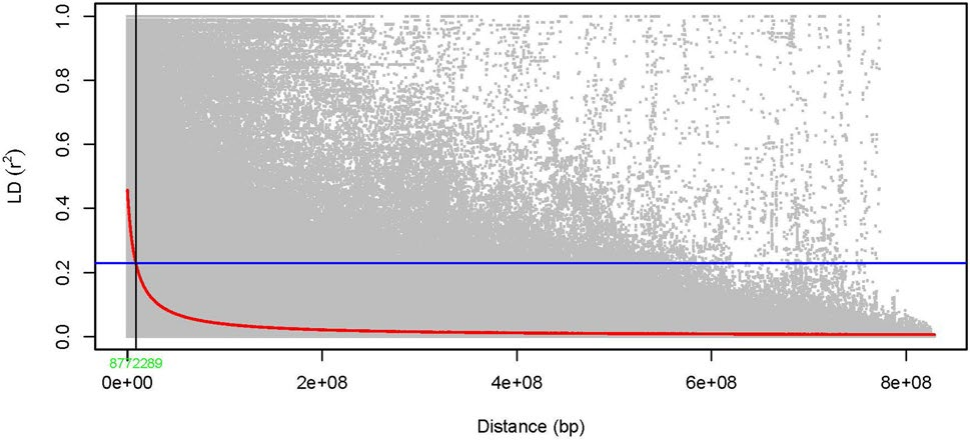
Scatter plot for the pairwise SNPs genome-wide linkage disequilibrium (LD) decay measured for 252 elite wheat genotypes in ICARDA. Genetic distance in Mbp is plotted against pairwise LD r^2^ values. The red solid curve represents the genome-wide LD decay generated from the smoothing spline regression model. The horizontal blue line represents the half-LD decay e (*r*^2^ = 0.23), and the vertical black solid line represents the genetic distance (8.77) at which the half-LD decay intersects with the LD decay curve

The linkage disequilibrium analysis exhibited the D sub-genome with the highest linkage disequilibrium having an average *r*^2^ value of 0.21 followed by the sub-genomes B and A with 0.20 and 0.19, respectively. The genome-wide linkage disequilibrium revealed the LD decay started at *r*^2^ value of 0.46. The half-LD decay (*r*^2^ = 0.23) intersected with the LD decay trend at 8.77 Mbp (Fig. 4).

**Fig. 5:**
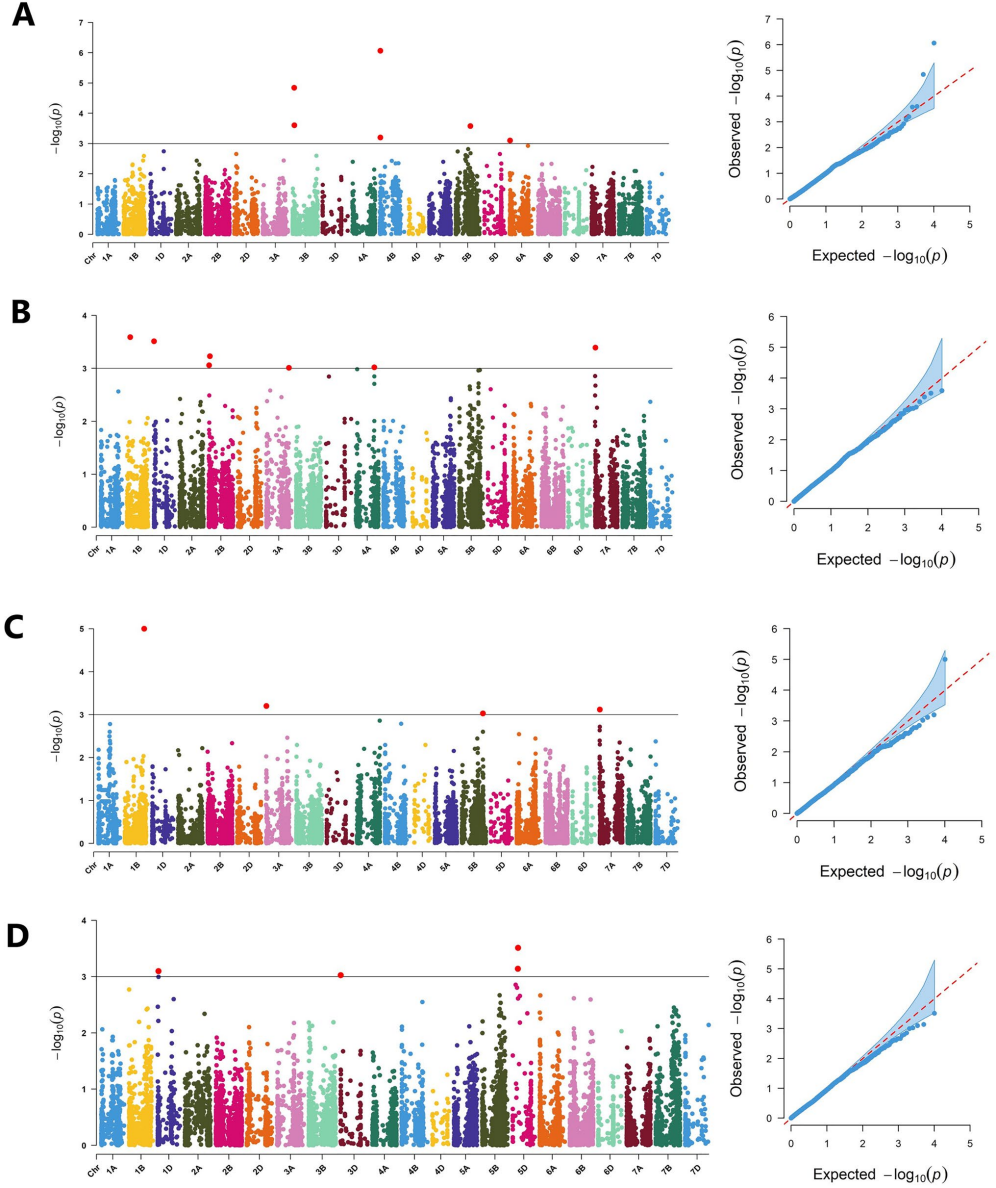
Manhattan (left) and Q–Q (right) plots for grain yield (**A**), Fe (**B**), Zn (**C**), and Se (**D**) traits identified through GWAS analysis. The horizontal solid line indicates the exploratory significant threshold applied at *P* of 0.001 or −log*P* < 3 to identify MTAs

A total of 19 marker-trait associations (MTAs) was identified significantly (*P* < 0.001) linked to the 4 tested traits (Table 2). Six MTAs were identified as significantly linked to grain yield under drought condition. The marker *IAAV2271* located on chromosome 4B was particularly highly significantly (*P* < 0.000001) linked with grain yield with −log_10_^P^ value of 6.06 followed by the markers *BobWhite_c4502_252* and *RAC875_c46194_201* which are both located on chromosome 3B with −log_10_^P^ value of 4.8 and 3.6, respectively. Other six MTAs were identified for Fe located on chromosomes 3A, 4A, 7A, 2B, and 1D (Table 2, Fig. 5). *BS00049927_51* located on chromosome 1B was the SNP marker identified as highly significantly linked with Fe with −log_10_^P^ value of 3.58. For zinc content, four significantly linked MTAs were identified on chromosomes 3A, 7A, 1B, and 5B. Marker *RAC875_c66649_186* located on chromosome 1B was identified as significantly linked with Fe with −log_10_^P^ value of 5.0. The marker *Excalibur_c10046_579* on chromosome 5D was detected to be linked with selenium content.

**Table 2:** List of identified marker-trait associations significantly linked with Fe, Zn, Se, and grain yield identified through genome-wide association analysis

Trait	Marker	Chromosome	Position (bp)	*R*^2^	–log10(*p*)
Fe	*BS00049927_51*	1B	108,837,029	0.06	3.58
Fe	*wsnp_Ku_c4299_7814936*	7A	9,753,520	0.06	3.38
Fe	*BS00077914_51*	2B	42,281,519	0.06	3.22
Fe	*Kukri_rep_c92967_369*	2B	15,882,612	0.05	3.05
Fe	*wsnp_Ex_c13031_20625900*	4A	604,209,019	0.04	3.01
Fe	*wsnp_Ex_c45877_51547406*	3A	743,190,499	0.05	3.00
Zn	*RAC875_c66649_186*	1B	626,707,491	0.09	5
Zn	*RAC875_c36922_829*	3A	9,852,634	0.04	3.19
Zn	*BS00079237_51*	7A	3,906,831	0.04	3.11
Zn	*Kukri_c41408_233*	5B	684,612,171	0.04	3.02
Se	*Excalibur_c10046_579*	5D	160,064,658	0.06	3.51
Se	*RAC875_rep_c70595_321*	5D	154,539,742	0.05	3.13
Se	*Kukri_c33670_506*	1D	20,056,459	0.04	3.09
Yield	*IAAV2271*	4B	17,469,995	0.11	6.06
Yield	*BobWhite_c4502_252*	3B	54,752,111	0.09	4.8
Yield	*RAC875_c46194_201*	3B	59,064,804	0.06	3.60
Yield	*wsnp_Ra_c26091_35652620*	5B	483,000,113	0.05	3.57
Yield	*RAC875_c12959_869*	4B	17,469,492	0.06	3.19
Yield	*Ra_c6065_1145*	6A	9,327,369	0.05	3.09

A total of 16 putative candidate genes were discovered from the 19 identified SNP markers significantly associated to Fe, Zn, Se, and GY traits (Table 3). The marker *RAC875_c66649_186* highly significantly associated with Zn was linked with *TraesCS1B02G395000* gene which encodes for the zinc finger protein *BRUTUS* and is involved in zinc ion binding. The SNP marker *wsnp_Ku_c4299_7814936* identified on chromosome 7A had a significant association with Fe and is linked with the gene *TraesCS7A02G024800*. This gene encodes for the WAT1-related protein involved in transmembrane transporter activity. The SNP *Kukri_c33670_506* having a MTA with Se content from GWAS analysis is linked with the gene *TraesCS1D02G041400* which encodes for the Protein *gar2* involved in RNA binding. For grain yield, the marker *BobWhite_c4502_252* was linked to the gene *TraesCS3B02G086400*, which encodes for the *E3 ubiquitin-protein ligase* involved in the regulation of gene expression and ubiquitin-protein ligase activity.

**Table 3:** List of putative genes and their function identified from marker-trait associations for Fe, Zn, Se, and grain yield with the genome-wide association analysis

Trait	Gene	Protein name	Molecular function	Biological process
Fe	*TraesCS2B02G033000*	Tyrosine N-monooxygenase-like	Iron ion binding, monooxygenase activity	–
Fe	*TraesCS3A02G530000*	AP-3 complex subunit beta	–	Intracellular protein transport
Fe	*TraesCS4A02G313900*	Protein kinase domain-containing protein	Protein kinase activity	Protein phosphorylation
Fe	*TraesCS7A02G024800*	WAT1-related protein	Transmembrane transporter activity	–
Zn	*TraesCS1B02G395000*	Zinc finger protein BRUTUS	Zinc ion binding	Protein ubiquitination
Zn	*TraesCS3A02G013800*	Fatty acyl-CoA reductase	Alcohol-forming fatty acyl-CoA reductase activity	Long-chain fatty-acyl-CoA metabolic process
Zn	*TraesCS5B02G523800*	AAA domain-containing protein	Metalloendopeptidase activity	Proteolysis
Zn	*TraesCS7A02G008900*	Monocopper oxidase-like protein SKU5	Copper ion binding	–
Se	*TraesCS1D02G041300*	Mitoc_mL59 domain-containing protei	–	–
Se	*TraesCS1D02G041400*	Protein gar2	RNA binding	–
Se	*TraesCS5D02G118000*	HECT-type E3 ubiquitin transferase	Ubiquitin protein ligase activity	Protein ubiquitination
GY	*TraesCS3B02G086400*	E3 ubiquitin-protein ligase	Ubiquitin protein ligase activity	Regulation of gene expression
GY	*TraesCS3B02G089200*	F-box domain-containing protein	–	–
GY	*TraesCS4B02G024500*	Dirigent protein	Carbohydrate binding	Phenylpropanoid biosynthetic process
GY	*TraesCS5B02G299400*	Transcription factor 25	–	–
GY	*TraesCS6A02G019400*	Protein transport protein sec16	–	Golgi organization

## Genomic prediction and cross-validation analysis

**Fig. 6:**
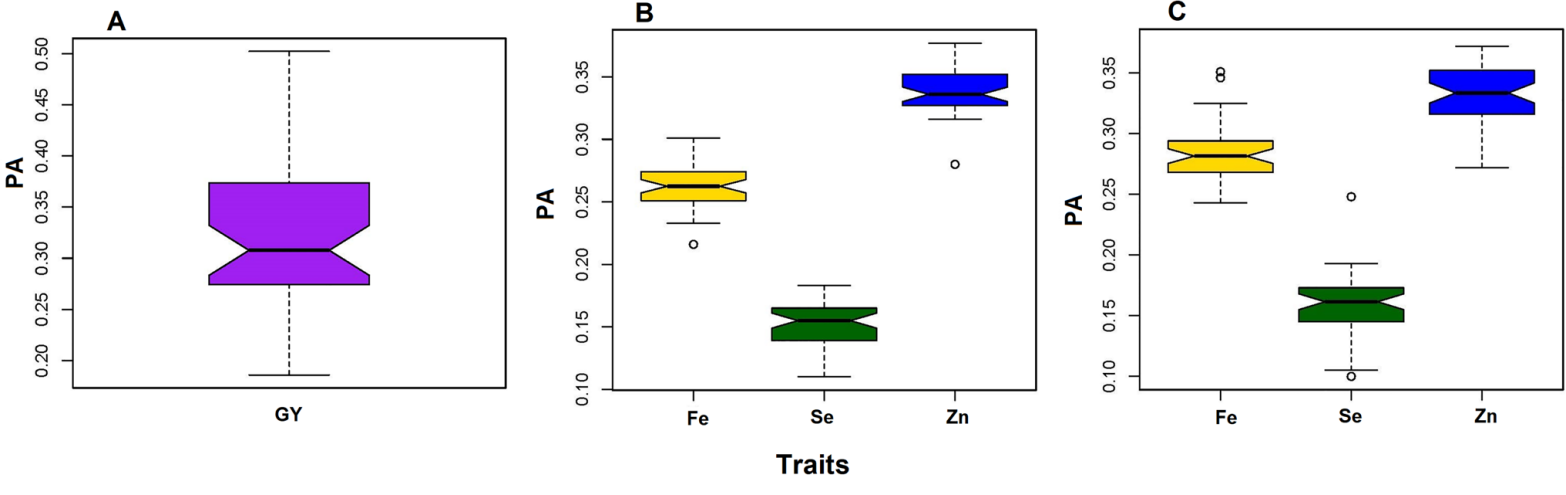
Predictive ability results from the single- and multi-trait-based genomic prediction analysis using the GBLUP model. The predictive ability for grain yield (**A**) was measured on a single-trait-based GBLUP model while the micronutrient-related traits were tested with both single-trait (**B**) and multi-trait (**C**) GBLUP models

The single-trait-based genomic prediction analysis of grain yield with the GBLUP model estimated an average predictive ability of 0.32 ranging from 0.186 to 0.502 (Table 4, Fig. 6). The genomic prediction for the other three micronutrient-related traits was explored with both single- and multi-trait-based genomic prediction methods in GBLUP model. The predictive ability of Se and Fe traits with multi-trait-based genomic prediction GBLUP model was 0.161 and 0.259 improving by 6.62 and 4.44%, respectively, compared to the single-trait-based models. However, the predictive ability of Zn remained constant in both methods (Table 4).

**Table 4:** Single- and multi-trait-based genomic prediction analysis for grain yield and micronutrient-related traits

Trait	Single-trait			Multi-trait		
	Minimum	Maximum	Average	Minimum	Maximum	Average
GY	0.186	0.502	0.32	–	–	–
Fe	0.216	0.301	0.248	0.243	0.35	0.259
Zn	0.28	0.379	0.341	0.278	0.378	0.34
Se	0.11	0.183	0.151	0.11	0.248	0.161

## Discussion

Grain yield and quality-related traits of crops are quantitatively inherited and are governed by many genes (polygenes). Environment, management, and the interaction between genotypes with these factors further affect the performance of wheat genotypes for these traits. The current study assessed the genotypic variation of 252 elite bread wheat genotypes for yield and micronutrients under rainfed conditions followed by GWAS and genomic prediction analysis. A significant variation was observed among evaluated genotypes for grain yield and micronutrient-related traits agreeing with several previous studies (Arora et al. 2019; Rathan et al. 2022; Gupta et al. 2022). According to Tadesse et al. (2015), the performance of yield and yield-related traits decreases under drought conditions while quality traits such as grain protein content and gluten content show significant increments. Agreed with previous studies, grain yield demonstrated a negative correlation with the currently studied quality traits (Kaya and Akcura 2014). In the current study, Fe exhibited a slightly significant positive correlation with Zn and Se inconsistent with previous reports (Bhatta et al. 2018). However, it is noteworthy to know that environmental factors play an important role in the performance of these traits and is important to consider both genotype and environment when making breeding decisions. The significant genotype x environment interaction further emphasizes the need to evaluate genotypes in multiple environments to identify those with broad adaptation.

GWAS has been used to identify unique MTAs and QTLs associated with valuable traits in different crops. In this study, we found a total of 19 significant markers associated with micronutrient-related traits and grain yield across different wheat chromosomes. Similarly, a higher number of MTAs was previously reported for grain yield, yield-related traits, and quality traits on the A and B genomes while the D genome relatively contributed less (Wang et al. 2021). We identified a highly significant marker for Fe content on chromosome 1B with −log_10_^P^ = 3.58 located at 108.8 Mbp. Several studies have detected many MTAs and QTLs for Fe content on the same chromosomes (Tong et al. 2022; Devate et al. 2022), but slightly on different positions indicating that these MTA could possibly be a novel marker discovered in our germplasm.

In this study, we identified four MTAs for Zn content located on chromosomes 1B, 3A, 5B, and 7A. The most significant marker was *RAC875_c66649_186* located on chromosome 1B. A previous study found the same marker on chromosome 1B linked to the grain filling rate (Yang et al. 2020), indicating that this marker is effective across different sets of materials tested in various environments and could be used for germplasm screening via MAS in breeding programs. In addition, we identified a marker highly associated with Se content on chromosome 5D with −log_10_^P^ = 3.51. Several MTAs and QTLs on chromosome 5D have been identified previously linked to Se under dry conditions (Ma et al. 2022). In this study, we found a total of six significant MTAs on chromosomes 3B, 4B, 5B, and 6A associated with grain yield. Previous studies have also reported MTAs linked to grain yield on the same chromosomes (Maphosa et al. 2014; Lesk et al. 2016; Li et al. 2018). Using a population of 127 RILs derived from the cross Ning7840 X Clark, Li et al. (2016) discovered the QTL QGpc.hwwgr-5BL on chromosome 5B.

Candidate genes linked to the MTAs with studied grain yield and micronutrient-related traits were mapped using the bread wheat genome reference database at Ensemble plant and UniProt. Previous studies have identified 16 genes involved in relevant biosynthetic pathways, including pathways related to zinc ion binding, Fe ion binding, ATP binding, monooxygenase activity, protein kinase activity, and ubiquitin-protein ligase activity (Raza et al. 2014; Michaletti et al. 2018). The candidate gene *TraesCS2B02G033000* located close to the marker *BS00077914_51*, which is associated with the Fe content, encodes for the *Tyrosine N-monooxygenase-like* enzyme which is involved in Fe ion binding and monooxygenase activity (Robertson and Biaggioni 2012). The marker *RAC875_c66649_186* located close to the gene *TraesCS1B02G395000* encodes for zinc finger protein BRUTUS and this protein is involved in zinc ion binding and protein ubiquitination (Rodríguez-Celma et al. 2019). *TraesCS4B02G024500* closely found with the SNP marker RAC875_c12959_869 is one of the five identified putative genes linked to grain yield. This gene encodes for Dirigent protein which is involved in carbohydrate binding and phenylpropanoid biosynthetic process (Li et al. 2019).

Genomic prediction can be a powerful tool for plant breeding and has the potential to significantly accelerate the development of new improved crop varieties (Alemu et al. 2023). The use of genomic prediction, genomic selection when the method applied practically in breeding, has been identified as the best strategy to increase the breeding efficiency of genetically complicated low-heritable traits in breeding (Alomari et al. 2018). Our genomic prediction results showed moderate prediction accuracies of mineral traits, which is consistent with previous studies that found low to moderate genomic predictability values of micronutrient-related traits (Kristensen et al. 2018; Mérida-García et al. 2019). The multi-trait-based genomic prediction methods could improve the accuracy of genomic selection, especially highly correlated traits, such as several micronutrient-related traits in crops (Atanda et al. 2022). Comparing the previous genomic prediction studies of micronutrient-related traits in wheat to other crops, rice shows a low to moderate prediction accuracy values (0.21 to 0.52) (Muvunyi et al. 2022) while a moderate prediction accuracy in maze (0.34 to 0.47) (Mageto et al. 2020). A previous genomic prediction study in pea quality traits shows low to moderate prediction accuracy (Atanda et al. 2022).

To conclude, the current study investigated the potential of ICARDA’s elite wheat genotypes for grain yield and three micronutrient-related traits followed by identification of MTAs and estimating the GEBVs of individual genotypes via GWAS and genomic prediction studies, respectively. Nineteen MTAs and sixteen associated putative genes were identified with the potential to facilitate the MAS process in the search for developing new improved varieties. The multi-trait-based genomic prediction analysis leads to an improved prediction accuracy for highly correlated micronutrient-related traits.

### Supplementary Information

Below is the link to the electronic supplementary material.

Supplementary file1 (XLSX 26 KB)

## Data Availability

The datasets generated for this study are included in the article and as supplementary files.
